# 1010. Cross-Species Translation of Correlates of Protection for COVID-19 Vaccine Candidates Using Quantitative Tools

**DOI:** 10.1093/ofid/ofab466.1204

**Published:** 2021-12-04

**Authors:** Anna Largajolli, Nele Plock, Bhargava Kandala, Akshita Chawla, Seth H Robey, Kenny Watson, Raj Thatavarti, Sheri Dubey, S Y Amy Cheung, Rik de Greef, Jeffrey R Sachs

**Affiliations:** 1 Certara, Princeton, New Jersey; 2 Merck & Co., Inc., Kenilworth, New Jersey

## Abstract

**Background:**

Several COVID-19 vaccines have been authorized, and the need for rapid, further modification is anticipated. This work uses a Model-Based Meta-Analysis (MBMA) to relate, across species, immunogenicity to peak viral load (VL) after challenge and to clinical efficacy. Together with non-clinical and/or early clinical immunogenicity data (ECID), this enables prediction of a candidate vaccine’s clinical efficacy. The goal of this work was to enable the accelerated development of vaccine candidates by supporting Go/No-Go and study design decisions, and the resulting MBMA can be instrumental in decisions not to progress candidates to late stage development.

**Methods:**

A literature review with pre-specified inclusion/exclusion criteria enabled creation of a database including nonclinical serum neutralizing titers (SN), peak VL after challenge with SARS-CoV-2 (VL), along with data from several clinical vaccine candidates. Rhesus Macaque (RM) and golden hamster (GH) were selected (due to availability and consistency of data) for MBMA modeling. For both RM and GH, peak post-challenge VL in lung and nasal tissues were used as surrogates for clinical disease and were related to pre-challenge SN via the MBMA. The VL predictions from the RM MBMA were scaled to incidence rates in humans, with a scaling factor between RM and human SN estimated using early Phase 3 efficacy data. This enabled clinical efficacy predictions based on ECID. To qualify the model’s predictive power, efficacies of COVID-19 vaccine candidates were compared to those predicted from the MBMA and their respective Ph1/2 SN data. More recently available clinical data enable building a clinical MBMA; comparing this to the RM MBMA further supports SN as predictive.

**Results:**

The MBMA analyses identified a sigmoidal decrease in VL (increasing protection) with increase in SN in all three species, with more SN needed (in both RM and GH) for protection in nasal swabs than in BAL (see figure). The comparison between predicted and reported clinical efficacies demonstrated the model’s predictive power across vaccine platforms.

RM and GH MBMA Protection Models and Translational Prediction with Observed Efficacies

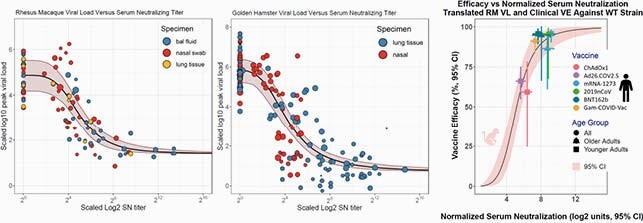

Sizes of circles indicate relative weight of the data in the respective quantitative model. Model and data visualizations have been harmonized (across tissue-types) separately for each of RM and GH using VACHER (Lommerse, et al., CPT:PSP, in press).

**Conclusion:**

By quantifying adjustments needed between species and assays, translational MBMA can inform development decisions by using nonclinical SN and VL, and ECID to predict protection from COVID-19.

**Disclosures:**

**Anna Largajolli, PhD**, **Certara** (Employee) **Nele Plock, PhD**, **Certara** (Employee, Shareholder)**Merck & Co., Inc.** (Independent Contractor) **Bhargava Kandala, PhD**, **Merck & Co., Inc.** (Employee, Shareholder) **Akshita Chawla, PhD**, **Merck & Co., Inc.** (Employee, Shareholder) **Seth H. Robey, PhD**, **Merck & Co., Inc.** (Employee, Shareholder) **Kenny Watson, PhD**, **Certara** (Employee, Shareholder) **Raj Thatavarti, MS**, **Certara** (Employee, Shareholder) **Sheri Dubey, PhD**, **Merck & Co., Inc.** (Employee, Shareholder) **S. Y. Amy Cheung, PhD**, **Certara** (Employee, Shareholder) **Rik de Greef, MSc**, **Certara** (Employee, Shareholder) **Jeffrey R. Sachs, PhD**, **Merck & Co., Inc.** (Employee, Shareholder)

